# Analyzing the role of ATP-dependent potassium channels in the regulation of coronary metabolic vasodilation during exercise

**DOI:** 10.1007/s00395-026-01179-4

**Published:** 2026-04-20

**Authors:** Salman I. Essajee, Cooper M. Warne, Johnathan D. Tune, Gregory M. Dick

**Affiliations:** Department of Physiology and Anatomy, University of North Texas Health Science Center, 3500 Camp Bowie Blvd., Fort Worth, TX 76107 USA

**Keywords:** Metabolic vasodilation, Functional hyperemia, KATP channels

## Abstract

The purpose of this meta-analysis is to compile, integrate, and assess studies that have investigated the role of ATP-dependent potassium channels (K_ATP_ channels) in coronary metabolic vasodilation in vivo. K_ATP_ channels are important modulators of membrane potential and vascular reactivity and are commonly proposed as regulating coronary blood flow in response to myocardial metabolism. For example, the electro-metabolic hypothesis of Lederer and colleagues (Zhao et al., Proc Natl Acad Sci 117:7461–70, 2020) suggests a pathway where myocardial metabolic activity reduces intracellular ATP, thus activating myocardial K_ATP_ channels to elicit vasodilation. The data collected here suggest that K_ATP_ channels are involved in determining resting vascular tone, but that their contribution does not increase with metabolism (a tonic 15% decrease in flow by glibenclamide). Furthermore, inhibiting K_ATP_ channels with glibenclamide impairs cardiac function by reducing coronary flow at rest, a clear indicator of ischemia, but this effect is overcome by endogenous mediators of vasodilation during increases in metabolism. These findings are inconsistent with the negative feedback/K_ATP_ channel version electro-metabolic hypothesis. Other cardiac K^+^ channels remain viable as candidates to mediate the vasodilatory mechanisms in this electro-metabolic paradigm. An amendment to the electro-metabolic hypothesis is proposed whereby the scheme is altered to feedforward. Specifically, we suggest considering the opening of other types of K^+^ channels in direct proportion to heart rate, ones whose activity can be linked directly to the increased frequency of cardiac action potentials or those that respond to mitochondrial factors or increased intracellular Na^+^. Whether the feedforward mechanism we propose can be supported experimentally remains to be determined.

## Introduction

Under physiological conditions, myocardial perfusion is tightly regulated to ensure myocardial oxygen delivery meets the oxidative requirements of ongoing cardiac contractile function. Coronary metabolic vasodilation comprises the mechanisms responsible for maintaining coronary blood flow in a linear relationship with the level of myocardial metabolism, such that increases in metabolic activity produce commensurate increases in coronary blood flow without over- or under-perfusion [1–6]. Vasodilation in response to ischemia (i.e., relative lack of blood flow) and hypoxia (i.e., relative lack of oxygen) are related, but mechanistically different, phenomena [7–10]. While the concept of perfusion-contraction-metabolism matching is simple in theory, understanding of how coronary blood flow is coupled to myocardial metabolism remains very poor [11, 12]. An exciting study in 2020 challenged the prevailing concept of coronary metabolic vasodilation and relies fundamentally on the activity of ATP-dependent potassium channels (K_ATP_) in cardiomyocytes [13, 14]. The electro-metabolic hypothesis of Zhao, Lederer, and colleagues suggests a pathway where myocardial metabolic activity reduces intracellular ATP, thus activating myocardial K_ATP_ channels to elicit vasodilation by two distinct mechanisms. First, K_ATP_ channel activation is proposed to hyperpolarize cardiomyocytes, and this voltage is transmitted to capillary endothelial cells, pericytes, and vascular smooth muscle cells to cause vasodilation. Second, the activation of K_ATP_ channels is proposed to increase the concentration of K^+^ in the interstitium, activate inwardly rectifying (K_ir_) K^+^ channels on other cells, and cause vasodilation. Thus, both mechanisms of vasodilation in the electro-metabolic hypothesis necessarily depend on K_ATP_ channels in cardiac myocytes.

K_ATP_ activity is sensitive to the metabolic state of the cell. These channels were first described as ATP-inhibited currents in cyanide-treated cardiomyocytes by Akinori Noma [15]. K_ATP_ channels are important regulators of vascular tone [16–19]. Since their discovery, the molecular nature, subunit composition, expression patterns, and function of K_ATP_ channels have been studied extensively. K_ATP_ channels can also be found in coronary endothelial cells [3] and coronary vascular smooth muscle cells [20]. Opening K_ATP_ channels (for example, with pinacidil) increases coronary blood flow [21]. Blocking K_ATP_ channels (for example, with glibenclamide) decreases coronary blood flow [21]. Further, the importance of K_ATP_ channels in regulating responses increases as vascular diameter decreases down the coronary arterial tree [22]. Thus, the anatomical location, inherent metabolic sensitivity, and pharmacological manipulation of K_ATP_ channels make them attractive candidates for study in the mechanisms regulating coronary blood flow.

A critical assessment of the electro-metabolic hypothesis and the role of K_ATP_ channels should include a thorough understanding of the effects of glibenclamide on coronary metabolic vasodilation in vivo. Specifically, a review of the literature should produce examples where glibenclamide antagonizes coronary metabolic vasodilation (i.e., disturbs the relationship between coronary blood flow and the level of myocardial metabolism). This is because glibenclamide blocks K_ATP_ channels in cardiac myocytes [23] and would be expected to attenuate cardiac myocyte hyperpolarization and interstitial K^+^ accumulation proposed in the electro-metabolic hypothesis. Glibenclamide also blocks K_ATP_ channels in coronary endothelial and smooth muscle cells [24]; therefore, data obtained with this pharmacological agent might be expected to demonstrate significant inhibitory effects on coronary metabolic vasodilation.

An important caveat for interpreting the role of K_ATP_ in coronary metabolic vasodilation is to restrict the analysis to metabolic stimuli [11, 12, 25–28]. That seemingly redundant statement might appear too obvious to justify its existence; however, discussions of coronary metabolic vasodilation are almost universally corrupted by introducing findings from studies that do not use a metabolic stimulus (i.e., exercise) to elicit a blood flow response. Rather, findings with primary stimuli such as hypoxia and ischemia are erroneously substituted without justification or consideration of differences in their underlying physiology (or more correctly, pathophysiology). Distinctly different mechanisms are involved in these other types of coronary vasodilation [7–10]. The purpose of this meta-analysis is to compile, integrate, and assess studies that have investigated the role of K_ATP_ channels in coronary metabolic vasodilation. The focus is on in vivo studies that used exercise as the stimulus for vasodilation. Given the strong correlation between heart rate and other determinants of myocardial oxygen consumption (MVO_2_; contractility, cardiac work) across species [11], we assessed the effect of K_ATP_ channel inhibition with glibenclamide on the relationship between coronary blood flow and coronary resistance relative to changes in heart rate at rest and during exercise.

## Methods

The National Library of Medicine PubMed database (https://pubmed.ncbi.nlm.nih.gov/) was searched for articles using the keyword combination “coronary metabolic vasodilation glibenclamide exercise”. The search returned 18 articles. These studies and the references within each were examined. The focus was narrowed to the 12 studies in Tables 1, 2, as our necessary criteria included: 1) measures of coronary blood flow, heart rate, and blood pressure; 2) exercise as the stimulus; and 3) the administration of glibenclamide. Coronary resistance was calculated by dividing mean blood pressure by the respective level of coronary blood flow. We collected data from published graphs as necessary with WebPlotDigitizer (https://automeris.io). Each data set was normalized to its respective baseline.

**Table 1:** Studies in dogs examining the effect of glibenclamide on exercise-induced coronary vasodilation

Study	Year	References	Glibenclamide dose	Data points/% Total
Billman et al	1993	[72]	10 mg/kg, i.v	14/18.7%
Duncker et al	1993	[21]	10 & 50 µg/kg/min, i.c	15/20.0%
Duncker et al	1995	[35]	50 µg/kg/min, i.c	10/13.3%
Duncker et al	1996	[34]	10 & 50 µg/kg/min, i.c	6/8.0%
Billman et al	1998	[46]	1 mg/kg, i.v	14/18.7%
Richmond et al	2000	[73]	1 mg/kg, i.v	8/10.7%
Chen et al	2001	[49]	50 µg/kg/min, i.c	8/10.7%

*i.v* intravenous, *i.c* intracoronary

**Table 2:** Studies in swine examining the effect of glibenclamide on exercise-induced coronary vasodilation

Study	Year	References	Glibenclamide dose	Data points/% Total
Duncker et al	2001	[74]	3 mg/kg, i.v	14/19.4%
Merkus et al	2003	[75]	3 mg/kg, i.v	14/19.4%
Merkus et al	2006	[76]	3 mg/kg, i.v	14/19.4%
Duncker et al	2022	[36]	50 & 100 µg/kg/min, i.c	18/25.0%
Tune et al	2025	[42]	3 mg/kg, i.v	12/16.7%

*i.v* intravenous, *i.c* intracoronary

In order to estimate the concentrations of free glibenclamide in the blood (Fig. 5A), several assumptions were made. Our calculations were performed using: a) blood as the volume of distribution for i.v. studies (assume 8% of body weight; 80 ml/kg) or known blood flow/min for i.c. studies; b) the known amount of glibenclamide infused (bolus for i.v. and steady infusion per minute for i.c); and d) 99% protein binding of glibenclamide [29].

Data were plotted and analyzed in GraphPad Prism (https://www.graphpad.com/). Multiple linear regression analysis was used to compare slopes of relationships based on average data provided from each respective publication. If slopes were equivalent, subsequent analysis of covariance (ANCOVA) was used to test for differences in elevation in the relationship between specific response (y-axis) variables relative to changes in the relevant independent (x-axis) variable. Nonlinear regression analyses were 2nd order. One sample t-tests were used to test data in Fig. 5 against a value of 100%. In all tests, the criterion for significance was set at P < 0.05.

## Results

**Fig. 1:**
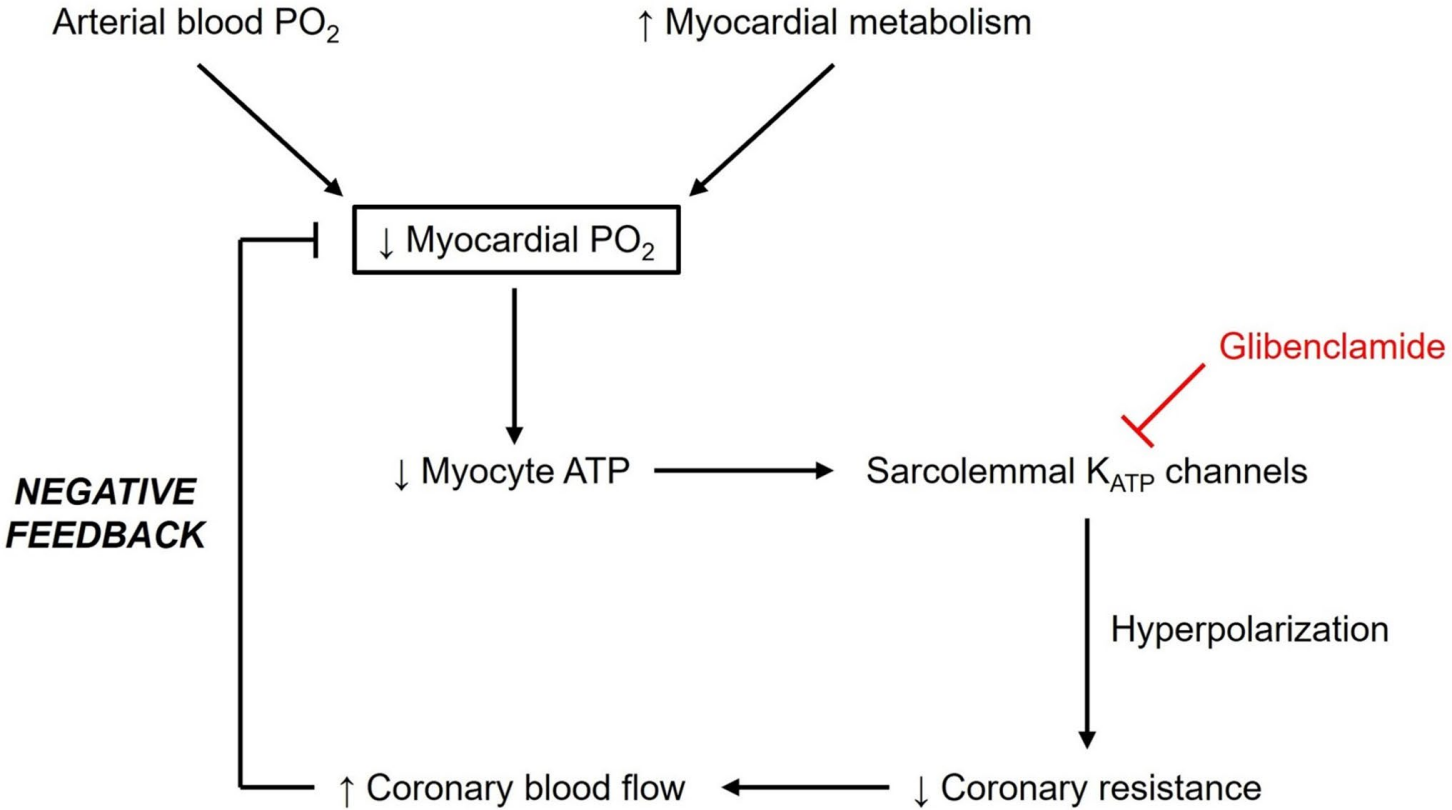
A simplified negative feedback diagram of the electro-metabolic hypothesis styled after the adenosine hypothesis [30, 31]. Myocardial PO_2_ (i.e., tissue oxygenation) is suggested to be the regulated variable. It has been proposed that as consumption of ATP by cardiomyocytes exceeds production, ATP levels fall, and K_ATP_ channels open. The K_ATP_ channels of cardiac myocytes are suggested to cause vascular hyperpolarization, increase coronary blood flow, and restore myocardial PO_2_ (and thus myocardial ATP). Because the K_ATP_ channels of cardiomyocytes are sensitive to block by glibenclamide, we surveyed the literature for effects of glibenclamide on coronary metabolic vasodilation in response to exercise, the primary physiological stimulus

Understanding the purpose of this paper requires comprehension of negative feedback regulation of coronary blood flow and our interpretation of the electro-metabolic hypothesis of coronary metabolic vasodilation ([14]; Fig. 1). Negative feedback regulation of coronary blood flow, in theory, is meant to balance myocardial oxygen delivery with oxygen consumption by adjusting blood flow in response to changes in a regulated variable (e.g., myocardial tissue PO_2_). The electro-metabolic hypothesis proposes that as consumption of ATP by cardiomyocytes exceeds production, ATP levels fall, and K_ATP_ channels open. The K_ATP_ channels of cardiac myocytes are suggested to hyperpolarize vascular cells, increase coronary blood flow, and restore myocardial PO_2_ (and thus myocardial ATP). A simplified negative feedback diagram of the electro-metabolic hypothesis styled after the adenosine hypothesis proposed independently by Berne [30] and Gerlach and colleagues [31] is shown in Fig. 1. Because the K_ATP_ channels of cardiomyocytes are sensitive to block by glibenclamide, we surveyed the literature for effects of glibenclamide on coronary metabolic vasodilation in response to exercise, the primary physiological stimulus. Our analysis was restricted to studies that measured coronary blood flow, heart rate and blood pressure, performed exercise, and included measures made with intravenous or intracoronary glibenclamide (Tables 1, 2).

**Fig. 2:**
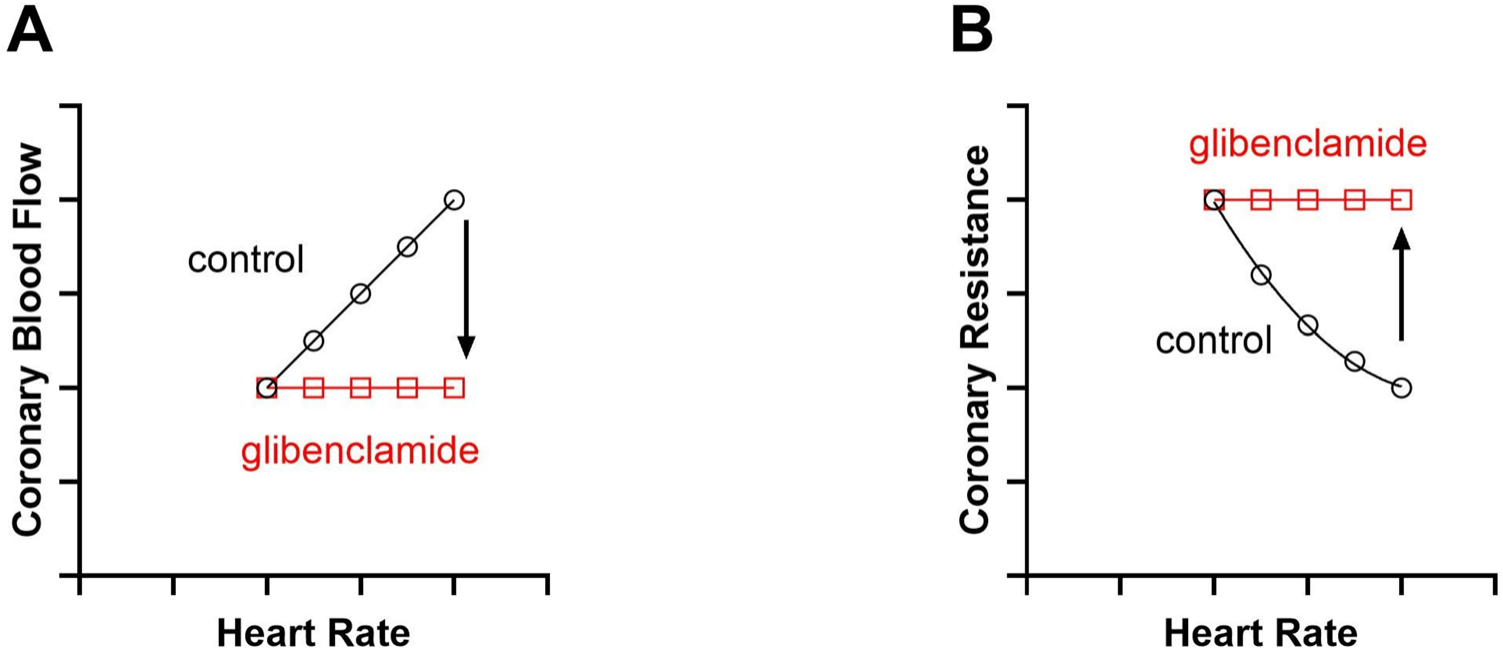
Predicting effects on coronary hemodynamics if glibenclamide were to block metabolic vasodilation. Panel A demonstrates that if K_ATP_ channels were responsible for coronary metabolic dilation, glibenclamide should prevent increases in coronary blood flow in response to exercise-mediated increases in heart rate. Panel B demonstrates that if K_ATP_ channels were responsible for coronary metabolic dilation, glibenclamide would prevent reductions in coronary resistance in response to exercise

Figure 2 shows predicted effects of glibenclamide on coronary hemodynamics if K_ATP_ channels were responsible for coronary metabolic vasodilation. Figure 2A demonstrates a strong linear relationship between coronary blood flow (alternatively, oxygen delivery) and heart rate with increasing exercise intensity. This is, by definition, metabolic coronary vasodilation. If glibenclamide were to completely inhibit coronary metabolic vasodilation, one would predict that coronary blood flow would be unchanged from baseline as heart rate increases with exercise (red line in Fig. 2A). Figure 2B highlights the curvilinear relationship between coronary vascular resistance and heart rate with increasing levels of exercise. If glibenclamide were to inhibit coronary metabolic vasodilation, coronary vascular resistance would remain unchanged (assuming no alterations in blood pressure) as heart rate is increased by exercise (red line in Fig. 2B; it should be noted that systolic extravascular compressive forces with increasing heart rate would actually increase coronary resistance). Figure 2 is drawn to show an extreme effect of glibenclamide. The data we collect could reasonably be expected to demonstrate partial block of coronary metabolic vasodilation by glibenclamide.

**Fig. 3:**
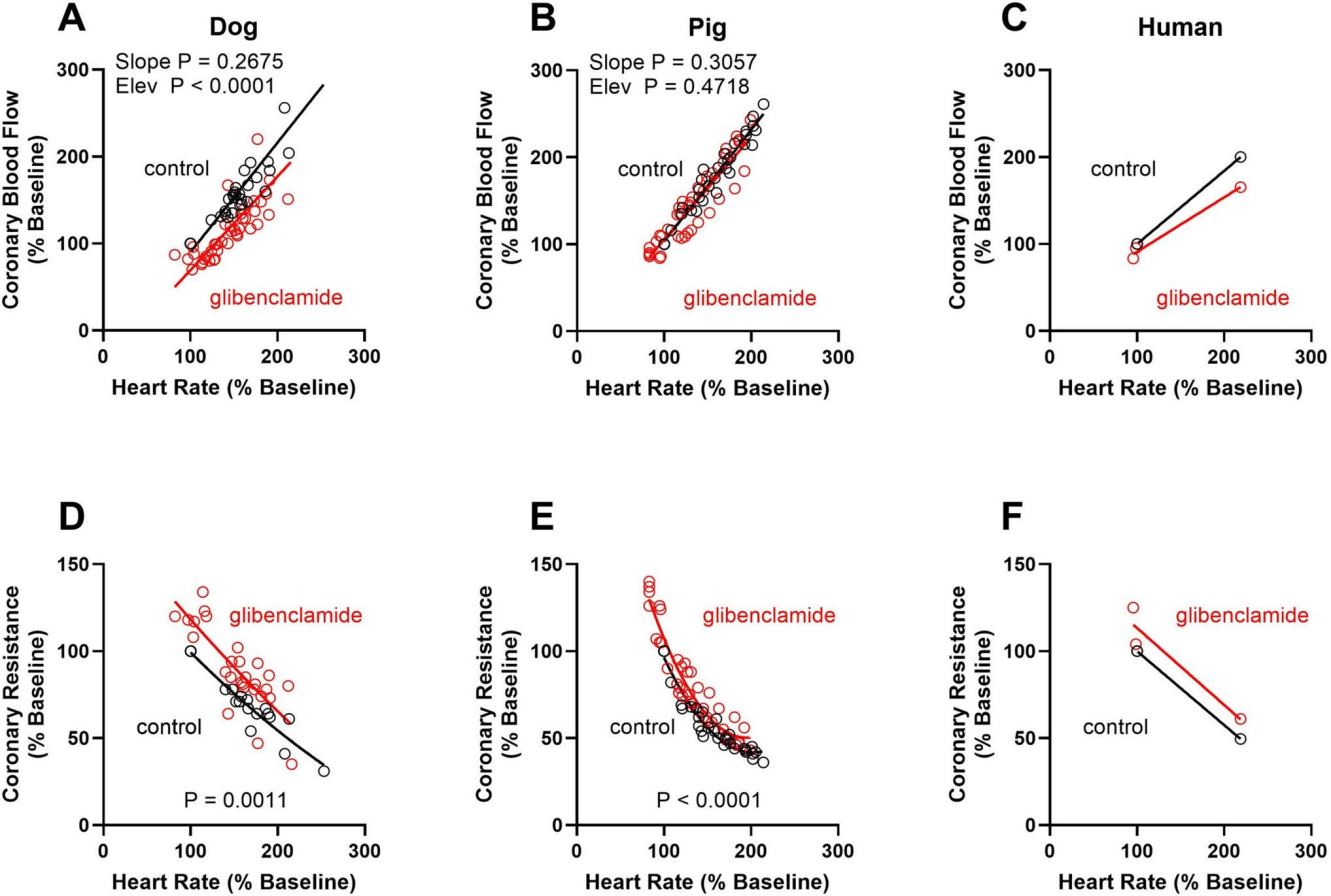
Experimental findings for the effects of glibenclamide on coronary hemodynamics during exercise. Panels A, B, and C illustrate the relationship between coronary blood flow and heart rate in dogs, pigs, and humans. Panel A demonstrates that glibenclamide decreased baseline coronary blood flow in dogs, resulting in a parallel downward shift in the relationship between coronary blood flow and heart rate during exercise. However, inhibition of K_ATP_ channels did not affect the slope of the relationship between coronary blood flow and heart rate, as would be predicted if K_ATP_ channels were involved in coronary metabolic vasodilation. Panel B illustrates that glibenclamide had no effect on the relationship between coronary blood flow and heart rate in pigs during exercise. Panel **C** contains data from humans regarding the relationship between coronary blood flow and heart rate during pacing (see Results for the details of statistical analyses performed by Farouque et al. [32, 33]). Panels D, E, and F show the relationship between coronary vascular resistance and heart rate in dogs, pigs, and humans

Figure 3 contains data collected from studies in Tables 1 (dogs) and **2** (pigs) showing the effect of glibenclamide on the relationships between coronary blood flow and coronary resistance relative to heart rate at rest and during exercise. Glibenclamide, whether administered intravenously or intracoronary, had a significant effect on the relationship between coronary blood flow and heart rate in dogs (Fig. 3A). Specifically, inhibiting K_ATP_ channels depressed the elevation of the relationship (P < 0.0001), but did not alter the slope (P = 0.2675). In exercising swine (Fig. 3B), glibenclamide had no significant effect on the relationship between coronary blood flow and heart rate, as neither the slope (P = 0.3057) nor the elevation (P = 0.4718) were affected. Data from humans for the effect of intracoronary glibenclamide on the relationship between coronary blood flow and heart rate are shown in Fig. 3C [32, 33]. These data come with an important caveat for interpretation, as elevations in heart rate were pacing-induced, not a result of exercise. Regardless, these data serve as a useful comparison. Farouque et al. [32, 33] found that glibenclamide reduced coronary blood flow at rest by about 9% (P = 0.04) and reduced pacing-induced flow about 26% (P = 0.03). The effects of glibenclamide on the relationship between coronary vascular resistance and heart rate are shown in Figs. 3D (dogs) and 3E (pigs). Glibenclamide significantly increased coronary vascular resistance in dogs (P = 0.0011) and pigs (P < 0.0001). Glibenclamide also increased coronary vascular resistance in the pacing study of Farouque et al. ([32, 33]; P = 0.03; Fig. 3F). When the blood flow data from dogs, pigs and humans are combined, the resulting lines for control and glibenclamide are parallel (P = 0.4206 for slope and P = 0.0152 for elevation). This significantly different elevation reveals a tonic ~ 15% inhibition of coronary blood flow by glibenclamide observed across the span of heart rates.

**Fig. 4:**
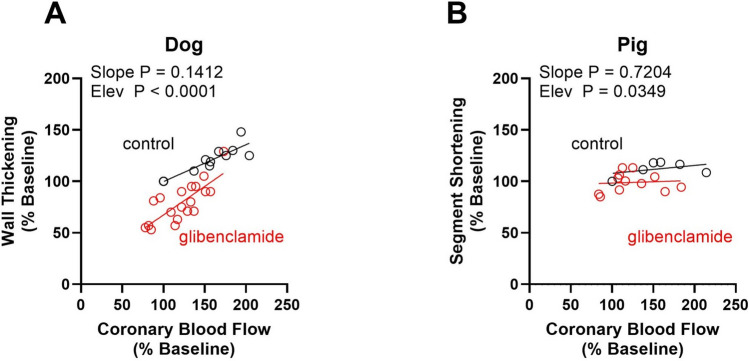
Experimental findings for the effects of glibenclamide on perfusion-contraction matching during exercise. Panel A contains data from studies in dogs [21, 34, 35] showing the relationship between wall thickening and coronary blood flow during exercise. Glibenclamide caused a significant parallel downward shift of the relationship between regional contractile function and coronary blood flow. Panel B contains data from swine showing the relationship between segment shortening and coronary blood flow during exercise. Glibenclamide caused a significant parallel downward shift of the relationship between regional contractile function and coronary blood flow

Figure 4 shows the effects of exercise on the relationship between regional contractile function and coronary blood flow in the absence and presence of glibenclamide in dogs (Fig. 4A, [21, 34, 35]) and swine (Fig. 4B, [36]). While these data represent all available studies, our assessment may be underpowered by the limited sample size available. Exercise-induced increases in regional systolic wall thickening in dogs were associated with linear increases in coronary blood flow (Fig. 4A). Consistent with the role of K_ATP_ channels in contributing to coronary flow regulation at rest, glibenclamide administration diminished regional contractile function in proportion to reductions in coronary blood flow. Inhibition of K_ATP_ channels depressed the relationship between coronary blood flow and contractile function in response to exercise in dogs (P = 0.1412 for slope and P < 0.0001 for elevation). That is, glibenclamide caused a significant parallel downward shift of the relationship between regional contractile function and coronary blood flow. The overall effect of K_ATP_ channel inhibition on the relationship between regional segment shortening and coronary blood flow in swine is shown in Fig. 4B. Inhibition of K_ATP_ channels decreased contractile function in response to exercise in swine (P = 0.7204 for slope and P = 0.0349 for elevation; therefore, a parallel downward shift of the relationship). Pigs increase cardiac output during exercise almost entirely by increasing heart rate, not stroke volume [37]; therefore, the relationship between segment shortening and heart rate is predictably very shallow.

**Fig. 5:**
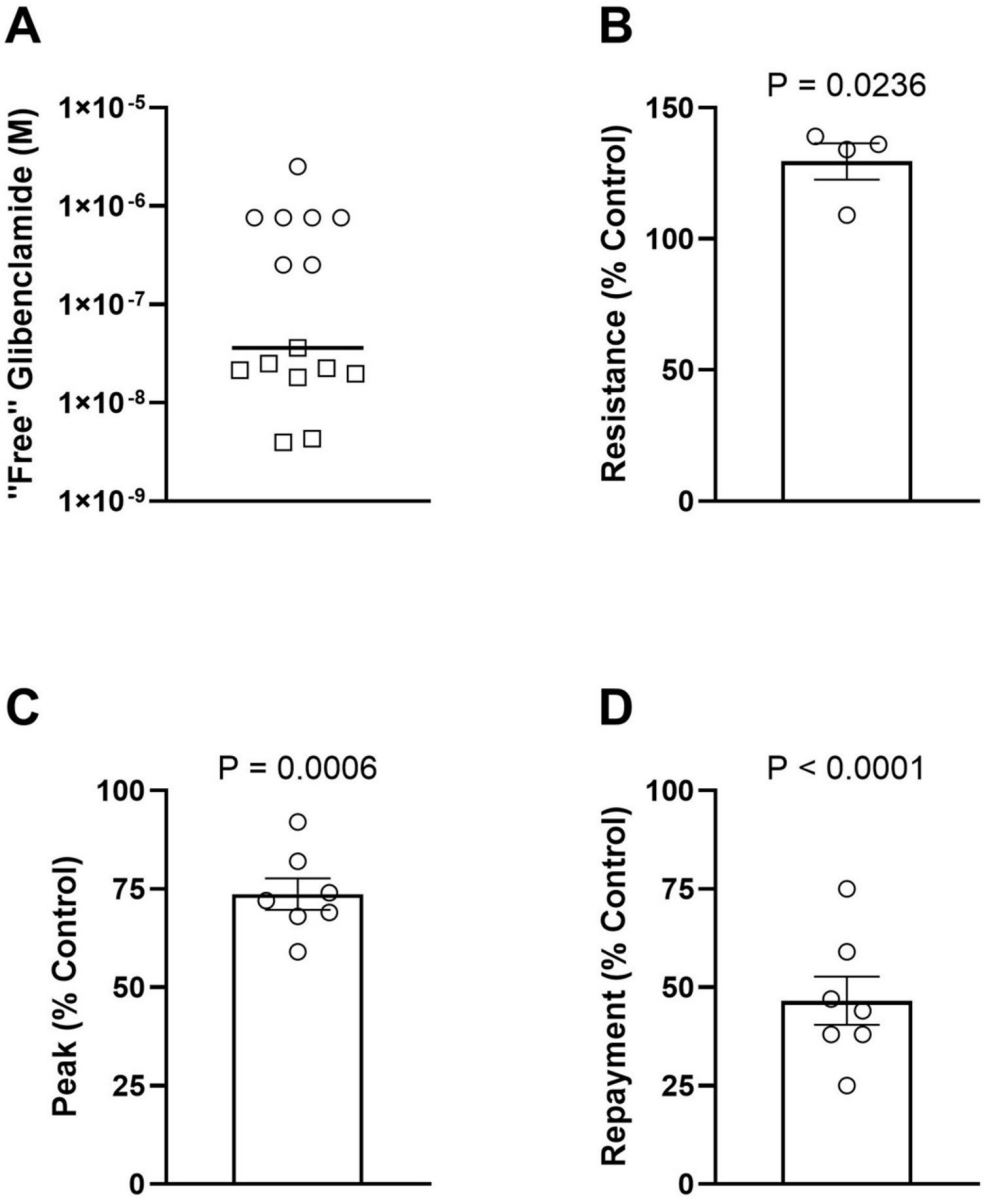
Estimated plasma concentrations of glibenclamide and their effects on reactive hyperemia and coronary vascular resistance. Panel A contains calculations of the estimated plasma concentration of glibenclamide from the studies in Tables 1, 2. Circle symbols represent i.v. doses, while square symbols represent i.c. administration. The calculation assumes that 99% of glibenclamide is bound to plasma proteins; therefore, the estimated “free” (unbound) concentration of glibenclamide is shown. Horizontal line indicates the median value of 36 nM; the mean value is 415 nM. Panel B shows the effect of intracoronary glibenclamide on coronary vascular resistance in swine when measured using servo-controlled constant coronary flow. Panel C shows the effect of glibenclamide on peak flow during reactive hyperemia (i.e., ischemic dilation) following 10–30 s of total coronary occlusion. Panel D illustrates the effect of glibenclamide on flow repayment following 10–30 s of total coronary occlusion

An expected and logical criticism of the analysis of these studies is that they all rely on the pharmacological effect of glibenclamide to inhibit K_ATP_ channels in vivo. Thus, several important questions must be addressed including the concentrations of glibenclamide used, the effectiveness of these doses, and what the drug target is. Regarding effectiveness, in all studies examined, glibenclamide treatment reduced coronary blood flow at rest. This finding clearly indicates that glibenclamide treatment in vivo effectively alters coronary vascular tone at rest. Regarding the site(s) of action for glibenclamide, this is impossible to determine from the existing data or experimental design. While K_ATP_ channels are undoubtedly a target of glibenclamide, the cell type affected is uncertain. There could be effects on coronary flow and metabolism secondary to insulin release from the pancreas. It could be interpreted that the increase in coronary vascular tone results from inhibition of K_ATP_ channels in cardiac myocytes, smooth muscle cells, endothelial cells, and/or mitochondria. Moreover, it is possible that glibenclamide has off-target effects that are unrelated to K_ATP_ channels (e.g., inhibition of metabolic enzymes [38]). The role of K_ATP_ channels in myocardial metabolism has been covered previously [39–41]. The estimated concentrations of glibenclamide and their effectiveness are demonstrated in Fig. 5.

Figure 5A shows the estimated concentration of “free” or “available” glibenclamide in the blood, assuming that 99% of it is bound to proteins and unable to exert pharmacological effects. The mean concentration is 415 nM (median 36 nM) and clearly reduces coronary blood flow at rest (Figs. 3 and 4). Figure 5B illustrates the effect of intracoronary glibenclamide on coronary vascular resistance in swine when measured using servo-controlled constant coronary flow [42]. Coronary vascular resistance is significantly increased with glibenclamide treatment. Thus, glibenclamide treatment in vivo is effective and has effects on vascular tone consistent with inhibition of K_ATP_ channels. Figure 5C shows the effect of glibenclamide on peak hyperemic flow (i.e., reflective of the minimum vascular resistance) achieved during ischemic dilation of 10–30 s duration [21, 35, 43–45]. Figure 5D shows the effect of glibenclamide on the repayment of flow debt incurred during 10–30 s of total coronary occlusion. These data clearly indicate that K_ATP_ channels are critical elements mediating the coronary vascular response to ischemia and support the electro-metabolic hypothesis in the context of ischemic dilation. It is difficult to conceptualize how glibenclamide is so effective at reducing resting coronary blood flow and antagonizing ischemic vasodilation in vivo and at the same time so ineffective at blocking exercise-induced coronary vasodilation without concluding that K_ATP_ channels play little to no role in metabolic vasodilation. Thus, it seems the mechanisms proposed in the electro-metabolic hypothesis may better describe mechanisms of ischemic vasodilation rather than beat-to-beat regulation of coronary vascular resistance coupled through exercise-induced changes in metabolism.

**Fig. 6:**
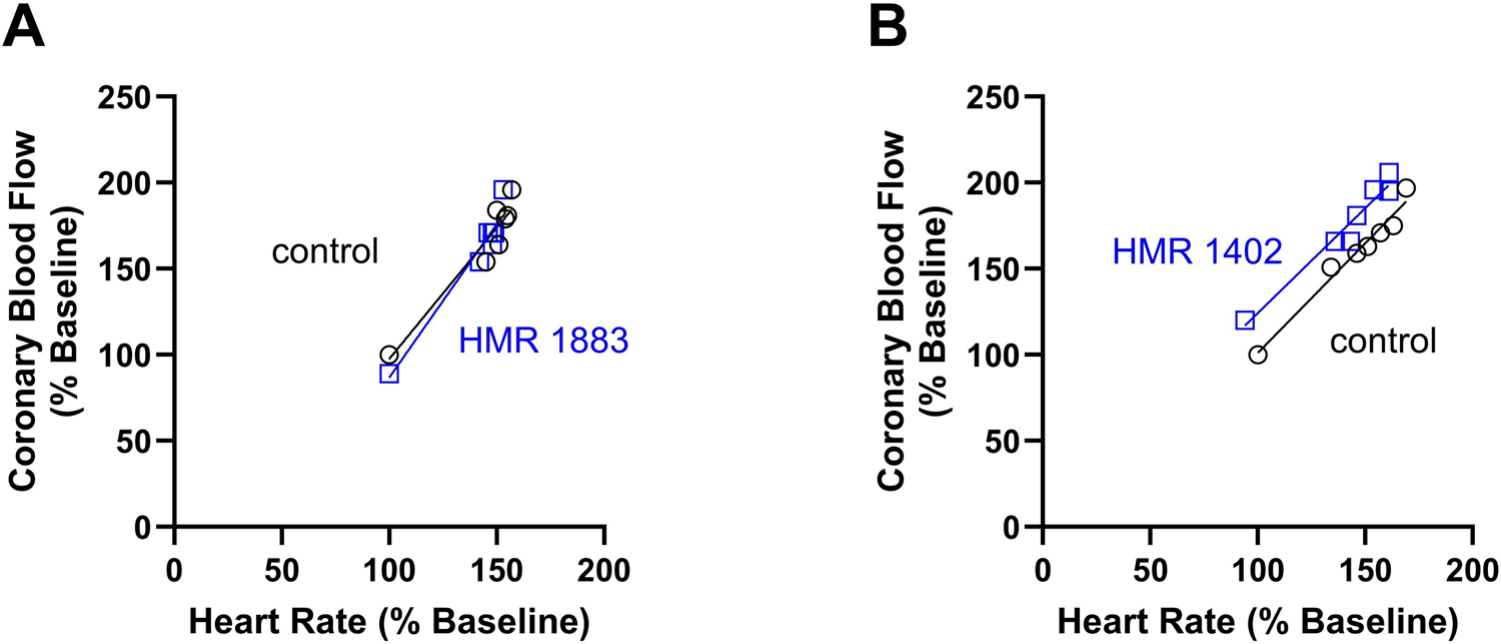
Coronary metabolic vasodilation during exercise with drugs that specifically target the K_ATP_ channels of cardiac myocytes. Billman and colleagues performed studies measuring coronary blood flow in exercising dogs treated with two cardio-selective K_ATP_ channel antagonists [46, 47]. The effects of HMR 1883 (Panel A) and HMR 1402 (Panel B) are shown. These novel pharmacological agents were reported to protect against ischemia-induced cardiac arrhythmias without compromising coronary blood flow, suggesting they indeed target myocardial K_ATP_ channels. Neither HMR 1883 nor HMR 1402 had an inhibitory effect on the relationship between coronary blood flow and heart rate during exercise

While the focus of this meta-analysis is on glibenclamide, our literature search revealed two studies [46, 47] with other sulfonylurea drugs designed to be more specific for targeting the K_ATP_ channels of cardiac myocytes (perhaps composed of Kir6.2 and SUR2A) than of those of vascular smooth muscle (perhaps composed of Kir6.1 and SUR2B). The molecular composition of mitochondrial K_ATP_ remains unclear, but this channel is also blocked by glibenclamide, and this has implications for coronary microvascular and cardiac function [20, 48]. Bache and coworkers demonstrated that glibenclamide inhibited myocardial oxygen consumption by reducing coronary blood flow (attributed to microvascular constriction), but that blocking mitochondrial K_ATP_ channels with 5-hydroxydecanoate had no effect on coronary blood flow or oxygen consumption [49]. Diazoxide, which opens K_ATP_ channels in mitochondria, has effects that mimic ischemic pre- and post-conditioning [50–52]. Two molecules purported to be selective for sarcolemmal K_ATP_ channels, HMR 1883 and HMR 1402 were demonstrated to have cardiac effects. Specifically, HMR 1883 and HMR 1402 shorten the myocardial action potential and reduce ischemia-induced arrhythmias [46, 47]. These drugs offer an interesting test of the electro-metabolic hypothesis, which fundamentally depends on the activity of cardiomyocyte K_ATP_ channels (Fig. 1). Neither HMR 1883 (Fig. 6A) nor HMR 1402 (Fig. 6B) reduced the relationship between coronary blood flow and heart rate. In fact, HMR 1402 modestly shifted upwards the relationship between coronary blood flow and heart rate. The reason is unclear, but HMR 1402 is reported to increase blood pressure [53]. A reasonable conclusion drawn from these studies would be that cardiac K_ATP_ channels are not critical elements mediating exercise-induced coronary metabolic vasodilation, which is in full agreement with the studies using glibenclamide (Figs. 3, 4). As mentioned above, the electro-metabolic hypothesis may better describe mechanisms that occur in response to ischemia than beat-to-beat regulation of coronary vascular resistance in response to the primary physiological stimulus of exercise.

**Fig. 7:**
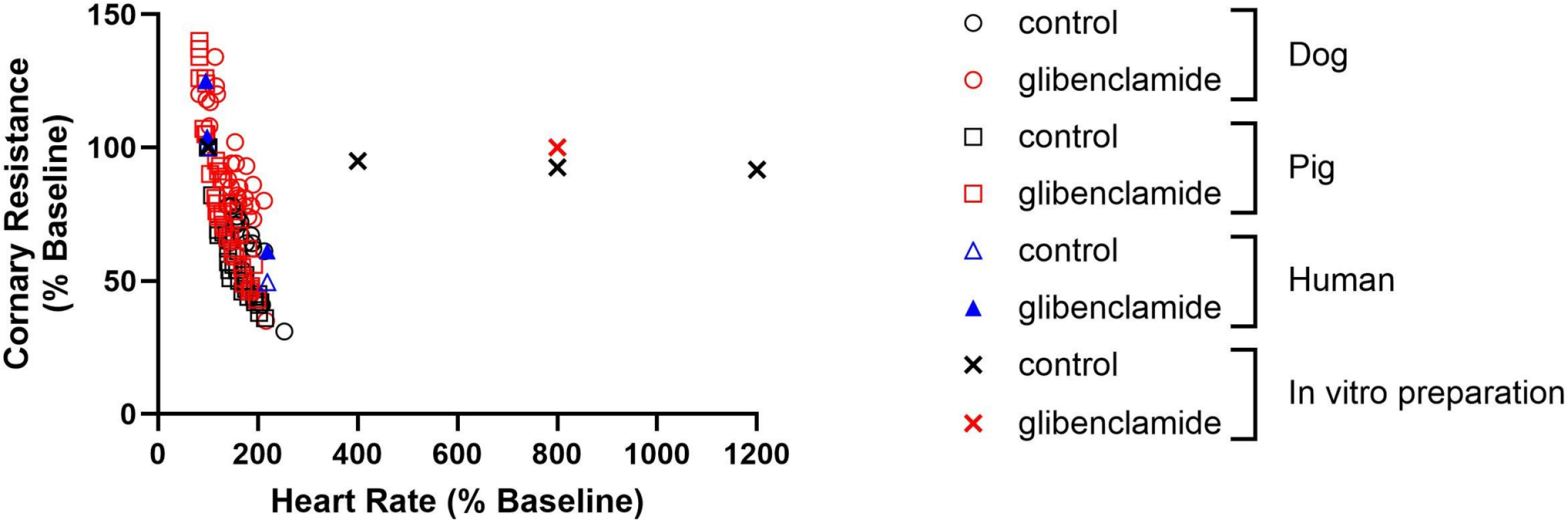
Comparing relationship between coronary vascular resistance and heart rate in exercising animals and paced humans vs. an isolated, cannulated myocardial preparation: Effect of glibenclamide. In vivo data were collected from the studies in Tables 1, 2 and the studies of Farouque et al. [32, 33]. In vitro data are from the paper by Zhao et al. describing the electro-metabolic hypothesis [14]. Two things are readily apparent from this comparison. First, the relationships between coronary vascular resistance and heart rate are vastly different between in vivo studies and the electrically paced myocardial muscle preparation. Second, the effect of glibenclamide on coronary vascular resistance is relatively minor both in vivo and in vitro

Misinterpretations of the electro-metabolic hypothesis may arise from the nature of the preparation, as it was an in vitro study of isolated cardiac muscle [14]. Specifically, rat and mouse ventricular muscle was perfused with crystalloid buffer. This preparation is, by definition, totally ischemic, as blood flow is zero. Further, while aerated with 20% oxygen, the preparation might also be hypoxic [54, 55]. Because of the definitive ischemia present in the isolated cardiac muscle preparation, there is almost no vascular tone and resistance changes very little with large increases in rate work (Fig. 7). Figure 7 compares the relationship between coronary vascular resistance and heart rate in exercising animals and paced humans vs. the pacing rate in the isolated, cannulated myocardial preparation. Further, the effect of glibenclamide on coronary vascular resistance is shown for in vivo and in vitro preparations. In vivo data were collected from the studies in Tables 1, 2. Data from humans were from by Farouque et al. [32, 33]. A very steep relationship between coronary vascular resistance and heart rate is obvious for in vivo studies. Those in vivo data truly represent coronary metabolic vasodilation, as coronary vascular resistance falls 50% under control conditions as heart rate increased. Glibenclamide increased coronary vascular resistance at rest by 24%, indicating coronary vasoconstriction. Importantly, however, in the presence of glibenclamide, coronary vascular resistance fell 49% as heart rate increased. Thus, the glibenclamide-induced increase in resistance was overcome by mediators of metabolic coronary vasodilation. The i*n vitro* data are from the paper by Zhao et al. describing the electro-metabolic hypothesis [14]. The relationship between vascular resistance and pacing rate is extremely shallow (i.e., an 8% change) across a ten-fold change in heart rate. If one focused only on pacing frequencies that might reasonably translate to resting and exercising heart rates in rats (6 to 9 Hz), the fall in vascular resistance is only about 1%. Further, the effect of glibenclamide on coronary vascular resistance is relatively minor in the isolated muscle preparation (only about 1/3 of what was observed in vivo). It is difficult to conclude from these findings that glibenclamide-sensitive K_ATP_ channels mediate coronary metabolic vasodilation.

## Discussion

The data presented here suggests that the electro-metabolic hypothesis may better describe ischemic or hypoxic coronary vasodilation, rather than metabolic vasodilation. It does not appear to describe a mechanism that couples myocardial metabolism to coronary blood flow on a beat-to-beat basis during exercise-mediated increases in heart rate and myocardial work. The electro-metabolic hypothesis possesses elements which are truly paradigm-shifting in the field of coronary vascular regulation. Specifically, the idea that cardiac myocytes may electrically couple to vascular cells to signal for vasodilation is full of enormous research possibilities. This undeniably novel idea of Lederer and colleagues may reveal many new mechanisms and fundamentally improve our understanding of blood flow control. At present, however, the electro-metabolic hypothesis with a focus on K_ATP_ channels fails to withstand scrutiny as a fundamental mechanism of coronary metabolic vasodilation. The data in Fig. 3 regarding the relationships between coronary blood flow or coronary vascular resistance and heart rate indicate that glibenclamide-sensitive K_ATP_ channels are not obligatory for coronary metabolic vasodilation in response to exercise. Further, if the negative feedback scheme shown in Fig. 1 were viable, then the inhibitory effect of glibenclamide would be expected to increase during exercise (Fig. 2A). This is clearly not what is observed, as the antagonistic effect of glibenclamide on coronary blood flow and resistance at rest remains essentially unchanged as exercise intensity increases (Fig. 3; a tonic ~ 15% inhibition of coronary blood flow by glibenclamide was observed across the span of heart rates). Importantly, K_ATP_ channels may be responsible, at least in part, for dilation in response to ischemia (Fig. 5) and the electro-metabolic hypothesis may be unveiling these mechanisms during a time when myocardial ATP levels are known to fall [56–58] and the myocardial interstitial potassium concentration is recognized to increase [59–62]. There is great value in further understanding the pathophysiological mechanisms of coronary dilation.

Any metabolic hypothesis of coronary flow regulation via ATP and/or adenosine requires a decrease of ATP and increased adenosine production during exercise. These issues have been addressed previously (e.g., no increase in interstitial adenosine during exercise [11, 12, 63]), but the issue for ATP is not yet settled. While no in vivo study has demonstrated a decrease of myocardial ATP during exercise, there is exciting evidence emerging from Santana and coworkers [64] regarding the concentration of ATP in isolated, beating cardiomyocytes. Rhana et al. use a fluorescent sensor to measure ATP and demonstrated that the pool of freely available ATP is much lower than previously estimated (i.e., not 10 mM, but closer to 1 mM) and changes approximately 50% during contraction and relaxation [64]. This has important implications for the activity of K_ATP_ channels in cardiac myocytes and would support the electro-metabolic hypothesis. Considering the data presented here (Figs. 3, 4, and 6), however, it remains difficult to envision the K_ATP_ channel as the critical element in coronary metabolic vasodilation.

**Fig. 8:**
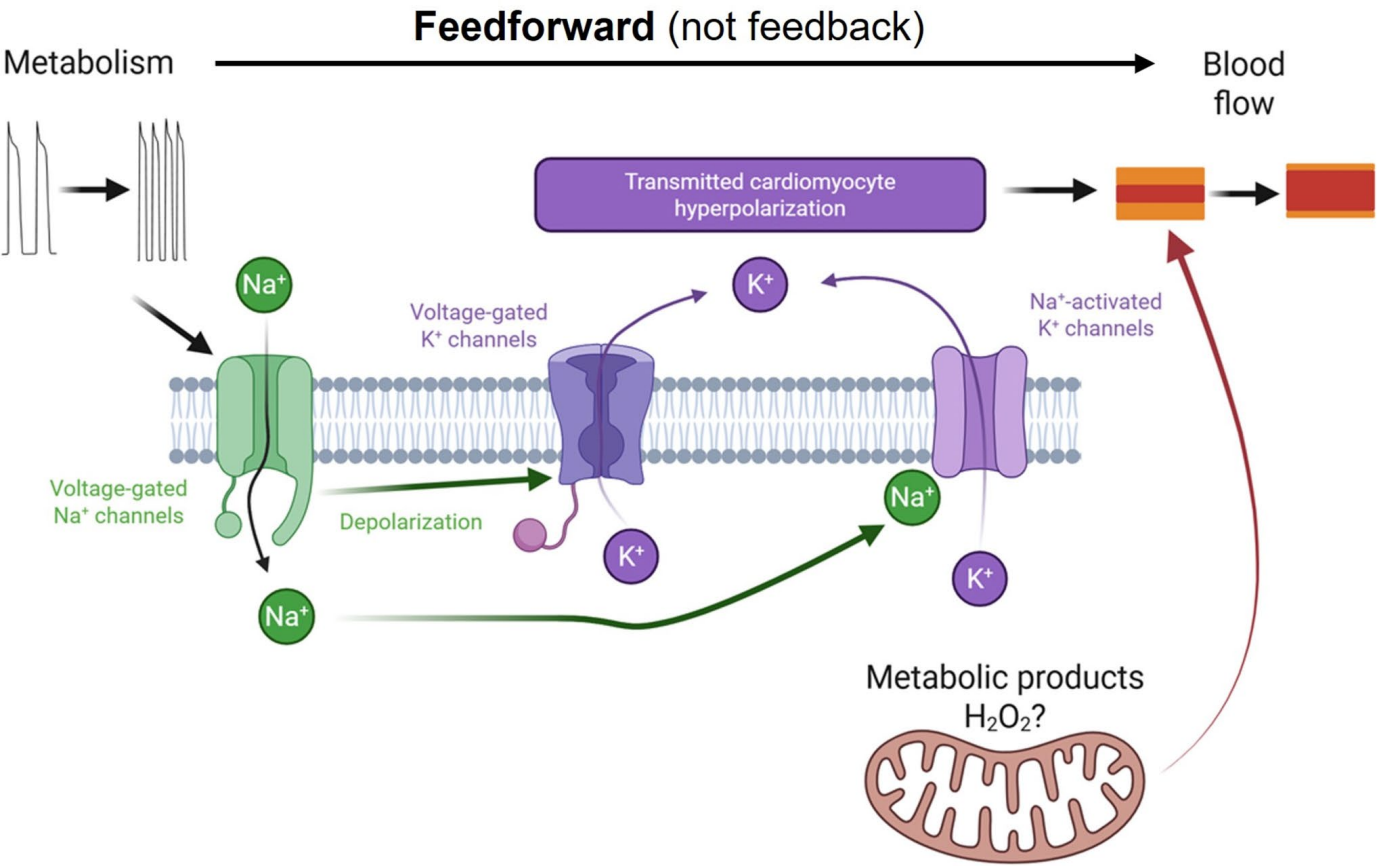
Proposed hypothetical feed-forward revision of electro-metabolic coupling Myocardial K^+^ channels that may serve as candidates in feed-forward electro-metabolic coupling. The idea presented here is that a lockstep relationship between myocardial metabolism and coronary blood flow exists and is controlled by a feedforward mechanism. Specifically, the idea that myocardial PO_2_ is the variable regulated by negative feedback is removed. The hyperpolarizing vasodilatory mechanisms of the electro-metabolic hypothesis are incorporated; however, they are suggested to rely on myocardial K^+^ channels other than K_ATP_ (denoted Kx). Of note is that other factors such as hydrogen peroxide may also play a requisite role in determining the degree of metabolic dilation to stimuli such as exercise. Hypothetically, coronary blood flow could be linked to metabolic demand (e.g., heart rate) through the cardiac action potential and influx of Na^+^. Each action potential is a membrane depolarization that activates voltage-gated K^+^ channels. The influx of Na^+^ during action potentials could stimulate Na^+^-activated K^+^ channels. The opening of voltage- and Na^+^-gated K^+^ channels could provide the hyperpolarization required as a mechanism in the electro-metabolic hypothesis. Another possible mechanism proposed is that the production of mitochondrial hydrogen peroxide (or other factors) may also be contributing to the observed vasodilation during exercise

A simple, but entirely untested, adjustment to the electro-metabolic hypothesis may realign it with mechanisms of coronary metabolic vasodilation during exercise (Fig. 8). Specifically, we propose changing from negative feedback based on tissue oxygenation (Fig. 1) to a feedforward mechanism based on heart rate (Fig. 8). The idea is that the lockstep relationship between myocardial metabolism and coronary blood flow is controlled by the hyperpolarizing mechanisms in the electro-metabolic hypothesis of Lederer and coworkers. The pathway is suggested to rely on myocardial K^+^ channels other than K_ATP_, as this seems to play a small role that does not change with exercise. Hypothetically, coronary blood flow could be linked to metabolism through heart rate, as the influx of Na^+^ could hyperpolarize by activating Na^+^-sensitive K^+^ channels that were first discovered in cardiac myocytes [65]. This proposal is not without its own difficulties, as increasing the intracellular Na^+^ concentration would have numerous consequences including changing Na^+^-dependent membrane transport (e.g., Na^+^/Ca^2+^-exchange) and altering electrical activity. Other possibilities remain within the plethora of voltage-dependent and -independent K^+^ channels found in cardiac myocytes. Additionally, because exercise is associated with an increase in sympathetic activity and β-adrenergic receptor activation, the adenylyl cyclase-protein kinase A pathway may increase the activity of myocardial potassium channels or the Na^+^/K^+^-ATPase that could lead to membrane hyperpolarization in a feed-forward manner. Finally, evidence for feedforward pathways factors produced in direct proportion to myocardial metabolism (e.g., H_2_O_2_) could also play a role (Figs. 8; [66]). Evidence suggests that K_ATP_ channels may be involved in feed-forward responses that resemble those mediated by the autonomic nervous system, as these channels are expressed on sympathetic nerve terminals and regulate norepinephrine release [67, 68]. Blockade of these channels, however, appears to increase rather than decrease the release of norepinephrine, particularly under ischemic conditions.

An anticipated rebuttal to this critique of the role of K_ATP_ channels in the electro-metabolic hypothesis concerns differences in spatial resolution between the in vivo and in vitro studies. That is, one might argue that the in vitro studies examine a myocardial microenvironment, while the in vivo studies are at the level of organ blood flow and do not possess the sensitivity to reveal interactions and mechanisms at the cellular level. This is a real possibility, and it is clear that spatial and temporal heterogeneities in coronary blood flow exist [69]. Without restrictions on coronary flow, the oxygen supply to low-flow regions is sufficient to meet metabolic demand, while the oxygen supply to high-flow regions reflects a higher local demand rather than overperfusion [70, 71]. Thus, blood flow heterogeneity most likely reflects differences in aerobic metabolism. However, if oxygenation, intracellular ATP, and myocardial K_ATP_ channels comprise the local mechanism responsible for regulating flow, then why do these mechanisms fail to make flow uniform? That is, if myocardial PO_2_ were the regulated variable in a negative feedback scheme (Fig. 1), then what mechanisms allow for reduced myocardial PO_2_ in vascular domains that possess this electro-metabolic machinery? This is a difficult question that has no readily apparent answer, but it may be because coronary metabolic vasodilation is better described by a feedforward mechanism linked directly to metabolism. (Fig. 8). Unfortunately, no studies have addressed whether the activation or inhibition of K_ATP_ channels make myocardial perfusion more or less heterogenous, as this might address the existence of electro-metabolic microenvironments. It is abundantly clear from the in vivo studies that animals treated with glibenclamide can still exercise, increase coronary blood flow, and reduce coronary vascular resistance to the same degree. Thus, even if there are local microdomains where electro-metabolic coupling fails due to inhibition of myocardial K_ATP_ channels with glibenclamide, there is little functional consequence. This is the unparalleled advantage of using in vivo models to assess physiological relevance, regardless of concerns over spatial resolution. It is also abundantly clear from the in vivo studies that glibenclamide does reduce coronary blood flow at rest (Fig. 3) and attenuate ischemic coronary vasodilation (Fig. 5). It is our opinion, from the data examined here, that the electro-metabolic hypothesis is not a local mechanism critical for regulating coronary metabolic vasodilation; therefore, there would be little value in pursuing the discussion if it were to retreat into speculatively inaccessible microdomains. Rather, the electro-metabolic hypothesis more likely describes a global mechanism fundamental to controlling ischemic and hypoxic coronary vasodilation and deserves further study in that context.

## Data Availability

All data are available from published studies.
